# Case Report: Partial nephrectomy in primary renal sarcoma presenting as Wunderlich syndrome; a rare tumour with rare presentation managed atypically

**DOI:** 10.12688/f1000research.18698.1

**Published:** 2019-04-10

**Authors:** Ramanitharan Manikandan, Ketan Mehra, Lalgudi Narayanan Dorairajan, Rajesh Nachiappa Ganesh, Sreerag K. Sreenivasan, Rajeev Kumar

**Affiliations:** 1 Department of Urology, Jawaharlal Institute of Postgraduate Medical Education and Research (JIPMER), Pondicherry, 605006, India; 2 Department of Pathology, Jawaharlal Institute of Postgraduate Medical Education and Research (JIPMER), Pondicherry, 605006, India

**Keywords:** Renal Sarcoma, Wunderlich syndrome, Partial nephrectomy, Undifferentiated sarcoma.

## Abstract

Spontaneous retroperitoneal haemorrhage also called Wunderlich Syndrome (WS) may be caused by various aetiologies. One of the most common causes is renal tumour. Renal sarcoma is a rare cause of WS, and renal sarcoma in itself is a rare entity. In the era of nephron-sparing surgery, optimum management of primary renal sarcoma remains a dilemma as there are limited number of cases available in the literature. Nevertheless, radical nephrectomy remains the recommended treatment, keeping in mind the aggressiveness of the tumour. We report a case of primary undifferentiated renal sarcoma, which presented as WS, and which was managed by partial nephrectomy.

## Introduction

Wunderlich syndrome (WS) is a life-threatening medical emergency defined as spontaneous non-traumatic bleeding confined to the perinephric region ^1^. It is often characterised by Lenk’s triad consisting of acute flank pain, flank mass and hypovolemic shock ^2^. Various aetiologies, including benign and malignant conditions attributable to renal and extra-renal origin, have been reported to cause WS in the literature ^3^. We report a rare presentation of WS in a young woman secondary to an underlying renal sarcoma, which was managed by robotic assisted laparoscopic partial nephrectomy. Furthermore, this report aims to address the difficulties in preoperative diagnosis and concerns of partial nephrectomy in the setting of renal sarcoma.

## Case report

A 21-year-old woman presented to the emergency department with a history of severe left flank pain and giddiness. She did not report any history of trauma, but had a past history of hypertension. The patient’s pulse rate was 96/min (reference range: 60–80/minute), blood pressure was 90/60 mmHg (reference range: 120/80 mmHg), haemoglobin was 5 gm% (reference range: 12–15 gm%), and serum creatinine was 1.8 mg/dL (reference range: 0.5–1.1 mg/dL). Ultrasonogram revealed a large left perirenal hematoma of size 8x6 cm. Non contrast CT scan suggested an 8 cm lesion in the left kidney without any other information about the aetiology. The patient received blood transfusions and intravenous fluids.

**Figure 1.**
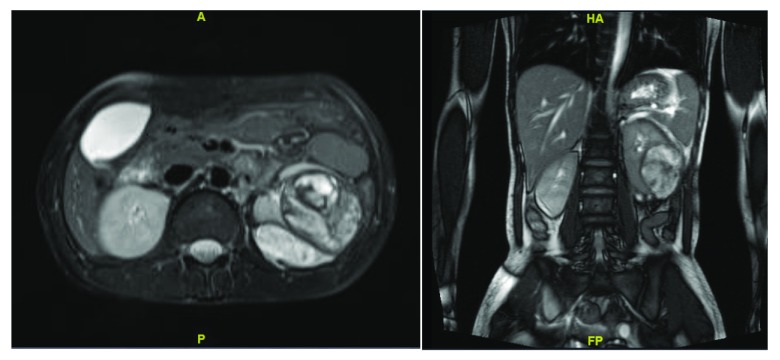
MRI abdomen coronal and transverse images depicting renal lesion in middle and lower pole of the left kidney.

**Figure 2.**
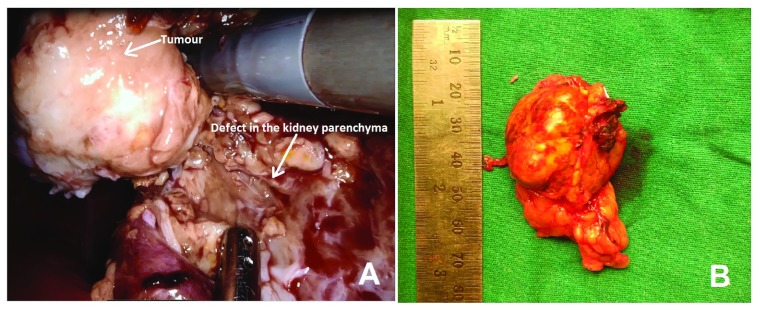
Robotic assisted laparoscopic left partial nephrectomy with guidance of intra-operative ultrasonography. ( **A**) Intraoperative image during partial nephrectomy of the left kidney; ( **B**) gross morphology of the resected tumour.

As the patient was stable, she was managed conservatively for the resolution of the hematoma to facilitate better surgical planning. After two weeks, the patient underwent MRI in which the hematoma had decreased in size to 5 cm, but the underlying cause could not be ascertained ( Figure 1). Keeping in mind that a renal tumour, either benign or malignant, could be the cause of haemorrhage, the patient underwent robotic assisted laparoscopic left partial nephrectomy with guidance of intra-operative ultrasonography to delineate the lesion margins ( Figure 2A). The warm ischemia time was 25 min with a blood loss of about 200 ml. The patient was discharged on day three with normal serum creatinine.

**Figure 3.**
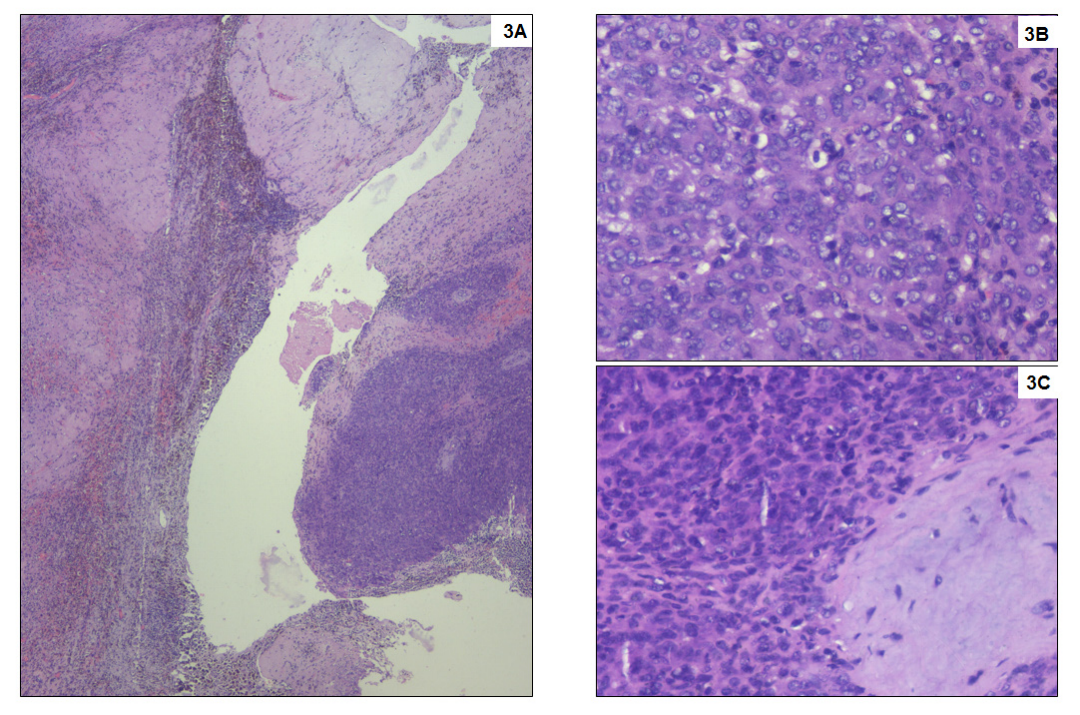
Haematoxylin and eosin staining of the tissue. ( **A**) Small round blue tumour cells arranged in sheets and nests infiltrating the adjacent stroma. Numerous hemosiderin laden macrophages are seen at the interface. No viable renal parenchyma is preserved, which is entirely replaced by dense fibrosis. Haematoxylin and eosin stain, x40; ( **B**) Tumour cell morphology at higher magnification with high nuclear cytoplasmic ratio, inconspicuous cytoplasm and occasional mitoses. Haematoxylin and eosin stain, x400; ( **C**) Tumour with adjacent bluish immature myxoid connective tissue. Haematoxylin and eosin stain, x400.

**Figure 4.**
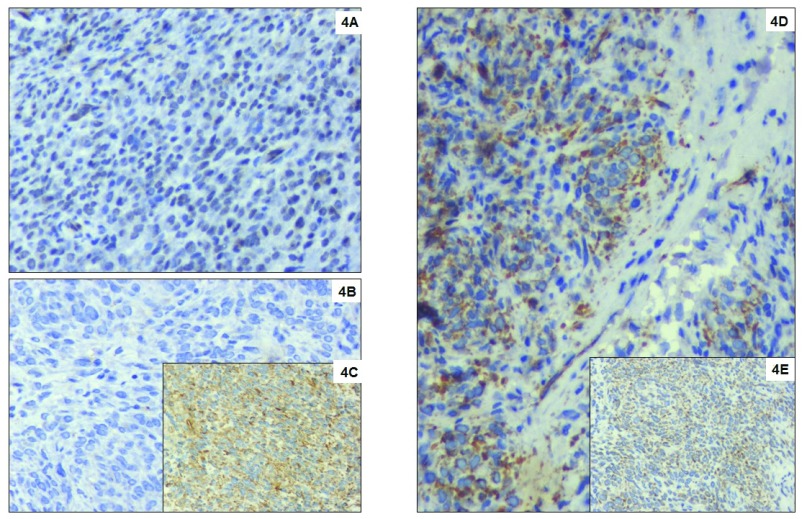
Immunohistochemical markers. ( **A**) Tumour cells with strong diffuse nuclear expression for FLI1. Immunohistochemistry with DAB counterstain, DAKO monoclonal antibody, x400; ( **B**) Negative staining of tumour cells with CD99. Immunohistochemistry with DAB counterstain, DAKO monoclonal antibody, x400; ( **C**) Strong diffuse staining of tumour cells for Vimentin. Immunohistochemistry with DAB counterstain, DAKO monoclonal antibody, x400; ( **D**) Strong cytoplasmic and Golgi expression of WT1 and no nuclear expression in tumour cells. Immunohistochemistry with DAB counterstain, DAKO monoclonal antibody, x400; ( **E**) Tumour cells showing strong cytoplasmic expression for Bcl2. Immunohistochemistry with DAB counterstain, DAKO monoclonal antibody, x400.

Macroscopically, the tumour was 5 cm in largest dimension, fleshy with yellow-brown appearance possibly due to haemorrhage ( Figure 2B). Microscopically, the tumour cells appeared small, round to oval shaped, with scant cytoplasm vesicular chromatin and tiny nucleoli with extensive haemorrhagic areas with hemosiderin laden macrophages. The tumour cells were about 5mm away from the resected margins. The tumour cells were positive for vimentin, BCL2 and FL1, and negative for Pan CK, CK7, CD10, CD31, S100, HMB45, SMA, ER, PR and WT-1, suggestive of “primary undifferentiated renal sarcoma” with hematoma ( Figure 3 and Figure 4).

**Figure 5.**
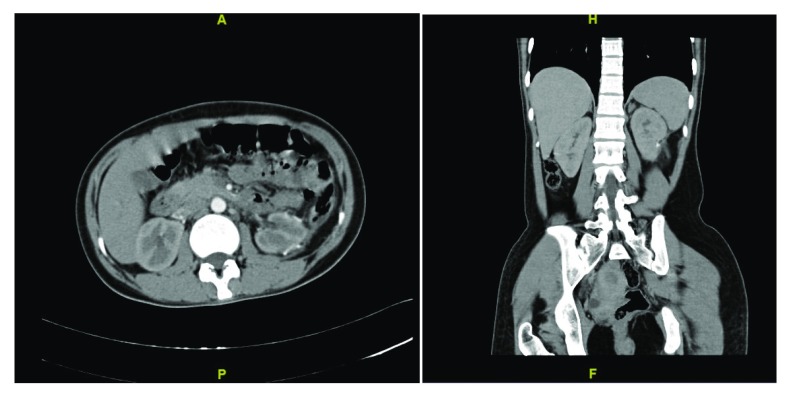
Follow-up contrast enhanced CT scan at 12 months showing no evidence of recurrence.

Since there are no definite guidelines regarding the role of partial nephrectomy in the background of renal sarcoma, we offered completion radical nephrectomy taking into account the young age of the patient. But the patient refused radical nephrectomy. Observation with regular follow-up was chosen by the patient as the best course of action. At the last follow-up of 12 months, there is no evidence of recurrence on contrast enhanced computerised tomography scan ( Figure 5).

## Discussion

In 1856, Wunderlich first described the condition of spontaneous renal bleeding with dissection of blood into the sub capsular and/or perinephric spaces ^1^. In a series of 165 patients with WS, Zhang *et al.* observed that renal neoplasm was the most common cause, with angiomyolipoma being the most common neoplasm ^4^. There are only scant reports of renal sarcomas presenting with primarily as WS ^5, 6^.

Sarcomas of the kidney constitute a heterogeneous group of rare neoplasms with aggressive clinical course and account for < 1% of renal cancers ^1^. Sarcomas usually present at a young age with a large tumour size. Renal sarcomas present with similar clinical and radiologic features as renal cell carcinoma and are rarely suspected pre-operatively ^7^. The 5-year overall survival rate for localised renal sarcoma is 46% in comparison to 8% for metastatic disease ^8^. As this tumour is rare, appropriate guidelines for the management and follow-up have not yet been established.

Moreira *et al.* performed a study with 489 patients of primary renal sarcoma. Primary treatment modality data was available for 367 patients; 210 (57%) underwent surgery, 51 (14%) underwent only radiation, 46 (13%) had both radiation and surgery, and 60 (16%) received neither radiation or surgery. The authors reported that surgery as the modality of treatment had a lower cancer specific mortality rate as compared to other measures. Nephron Sparing Surgery (NSS) or radical nephrectomy was not segregated in their study ^8^. The role of NSS in renal sarcomas is controversial. Wang *et al.* reported NSS in a patient with sarcoma with a disease-free interval of 42 months ^5^. Cocuzza *et al.* suggested that renal sarcomas with a diameter of 5 cm may be considered for NSS ^9^. Satoh *et al.* reported a case of cystic renal leiomyosarcoma, which was managed by partial nephrectomy with a normal follow up of 44 months ^10^. As long as the surgical margins are negative after NSS, it would be unnecessary to perform salvage radical nephrectomy in patients presumed to be renal cell carcinoma pre-operatively, but ultimately turns out to be a sarcoma in the final histology.

Our case presented as spontaneous renal haemorrhage at a young age. All three imaging modalities (ultrasonography, CT and MRI) were unable to delineate the cause of haemorrhage. In view of renal tumours being the most common cause, partial nephrectomy, through a minimal invasive approach, was accomplished. The final histopathology suggested it was a primary renal undifferentiated sarcoma. We were able to manage a case of renal sarcoma with nephron sparing surgery without disease recurrence on follow up. But due to lack of substantial literature, intense follow-up will be needed.

## Conclusion

Renal sarcoma presenting as Wunderlich syndrome is a rare phenomenon, which can mask its preoperative diagnosis. Due to scarcity of cases, optimum management of renal sarcoma is still debatable. Keeping in mind lack of evidence and aggressive nature of primary renal sarcoma, radical nephrectomy seems to be the optimum treatment. In the era of nephron sparing surgery, partial nephrectomy can be offered to patients of primary renal sarcoma with negative surgical margins.

## Consent

Written informed consent for publication of their clinical details and images was obtained from the patient.

## Data availability

All data underlying the results are available as part of the article and no additional source data are required.
