# Supplementary material for: Case Report: Partial nephrectomy in primary renal sarcoma presenting as Wunderlich syndrome; a rare tumour with rare presentation managed atypically
Source: F1000Res. 2019 Apr 10;8:423. [Version 1] doi: 10.12688/f1000research.18698.1 (PMC6652101; doi:10.12688/f1000research.18698.1)
Supplement: Peer review history

# Peer review: Reviewer response for version 1

**DOI:** 10.5256/f1000research.20474.r50127

**Published:** 2019-07-22

**Authors:** Hammad Ather

**Affiliations:** 1 Section of Urology, Aga khan University, Karachi, Pakistan

Authors have described a case of young woman presenting with spontaneous rupture of a renal sarcoma managed by RA partial nephrectomy. The management was unconventional as the standard approach to such a case is to manage conservatively as they did in the first few days, waiting for the resolution of hematoma and stabilization of the kidney function. Once the serum creatinine is normal she can be imaged either with contrast enhanced CT or MRU or contrast enhanced MRI. If the diagnosis is still uncertain a percutaneous biopsy and followed by definitive managment. The definitive managment, as the authors also pointed out is radical nephrectomy.

 The differential diagnoses included anaplastic sarcoma of the kidney (ASK), anaplastic Wilms tumor, mesenchymal chondrosarcoma, sarcomatoid renal cell carcinoma, clear cell sarcoma of the kidney, rhabdoid tumor of the kidney, congenital mesoblastic nephroma, and synovial sarcoma. The managment is highly dependent of the histology(Chen and Liao. Ci Ji Yi Xue Za Zhi. 2019 Apr-Jun;31(2):129-132 ^1^)

 My suggestions to the authors are:

1. Justify the indication of RA partial prior to establishing a diagnosis in a stable patient.
2. Discuss the histopathological aspects of the tumor and how to differentiate various types of sarcoma.
3. How the authors intend to follow up patient considering her young age. Is there a role of re biopsy of the tumor bed, for how long should the CT contrast or MRI scan be done. Are there any recommendations for just doing ultrasound if the CT or MRI are normal after first year.

I confirm that I have read this submission and believe that I have an appropriate level of expertise to confirm that it is of an acceptable scientific standard, however I have significant reservations, as outlined above.

## References

[1] : Anaplastic sarcoma of the kidney: Case report and literature review. *Tzu Chi Medical Journal*.2019;31(2) : 10.4103/tcmj.tcmj_194_18 10.4103/tcmj.tcmj_194_18 PMC645014931007495

# Peer review: Reviewer response for version 1

**DOI:** 10.5256/f1000research.20474.r46982

**Published:** 2019-07-08

**Authors:** Bipinchandra Pal

**Affiliations:** 1 Department of Urology and Transplantation Surgery, Institute of Kidney Diseases and Research Centre and Institute of Transplantation Sciences, Ahmedabad, India

- Little more clarification is needed in the radiological findings. 
- How was a plan decided for partial in presence of haematoma?
- Was a serum creatinine repeated before taking the case for surgery? Initially it was 1.8 mg% and after 3 days of surgery it was normal. Better to write the value.
- What is the follow up schedule?  

I confirm that I have read this submission and believe that I have an appropriate level of expertise to confirm that it is of an acceptable scientific standard.

# Peer review: Reviewer response for version 1

**DOI:** 10.5256/f1000research.20474.r46984

**Published:** 2019-04-30

**Authors:** Nikhil Khattar

**Affiliations:** 1 Department of Urology, Medanta, The Medicity, Gurugram, Haryana, India

Well written case report.

 It will be good if the authors can provide the NCCT picture at initial presentation with hematoma alongside the already provided MRI images.

 The authors should detail the follow up plan with duration considering the patients young age and propensity of renal sarcomas to recur even after 13-15 years ^1^.

 Are there any preoperative hints to suspect a sarcoma on imaging or with biomarkers published in the literature? A word about the same should find a place in the manuscript.

I confirm that I have read this submission and believe that I have an appropriate level of expertise to confirm that it is of an acceptable scientific standard, however I have significant reservations, as outlined above.

## References

[1] : Adult renal sarcoma: A rare case of recurrence 13 years after initial resection. *Clinical Case Reports*.2019;7(1) : 10.1002/ccr3.1911 47-50 10.1002/ccr3.1911 PMC633307630656006
